# Long-term golimumab persistence: Five-year treatment retention data pooled from pivotal Phase III clinical trials in patients with rheumatoid arthritis, psoriatic arthritis, and ankylosing spondylitis

**DOI:** 10.1007/s10067-023-06760-z

**Published:** 2023-09-26

**Authors:** Cindy L. J. Weinstein, Alan G. Meehan, Jianxin Lin, Steven D. Briscoe, Marinella Govoni

**Affiliations:** 1 Merck & Co., Inc, Rahway, NJ USA; 2 MSD Italy, Rome, Italy

**Keywords:** Golimumab, TNF-α, Treatment persistence, Rheumatoid arthritis, Psoriatic arthritis, Ankylosing spondylitis

## Abstract

**Introduction:**

Golimumab, a monoclonal antibody against tumor necrosis factor–α (TNF-α), is used widely for treatment of rheumatic diseases. Long-term persistence is an important factor influencing therapeutic benefit and is a surrogate measure of efficacy. We compared five-year golimumab treatment persistence across studies, indications, and lines of therapy using pooled data from pivotal golimumab Phase III clinical trials.

**Methods:**

This post-hoc analysis evaluated use of golimumab administered subcutaneously (50 or 100 mg every four weeks) for up to five years in 2228 adult participants with rheumatoid arthritis (RA; GO-BEFORE, GO-AFTER, and GO-FORWARD studies), psoriatic arthritis (PsA; GO-REVEAL study), or ankylosing spondylitis (AS; GO-RAISE study). Retention rate differences were evaluated by study, indication, and line of therapy using log-rank tests, and probability of treatment persistence was estimated by Kaplan–Meier analysis.

**Results:**

Golimumab retention rates at Year 5 were consistently high when used as 1^st^-line therapy (69.8%) and did not differ significantly across the three indications tested (*p* = 0.5106) or across 1^st^-line studies (*p* = 0.2327). Retention at Year 5 was better in participants using golimumab as 1^st^-line than in those using it as 2^nd^-line (41.6%) therapy. Participants on 2^nd^-line golimumab therapy had a longer disease duration (median 9.2 years versus 3.7 years) than those on 1^st^-line golimumab therapy.

**Conclusions:**

These data support the value of long-term golimumab therapy in patients with chronic, immune-mediated rheumatic diseases when used as 1^st^-line (RA, PsA, AS) or 2^nd^-line (RA) therapy.

**Table:** 

**Key Points** *• Golimumab is a human monoclonal antibody directed against tumor necrosis factor–α (TNF-α) and is approved widely for the treatment of rheumatic autoimmune diseases.* *• We compared the probability of treatment persistence, or the time of continuous drug use, for golimumab across five Phase III studies spanning multiple rheumatic indications over five years.* *• Treatment persistence was favorable and did not differ significantly for participants with rheumatoid arthritis, psoriatic arthritis, and ankylosing spondylitis, but persistence was greater when golimumab was used as 1st-line than as 2nd-line biologic therapy.*

**Supplementary Information:**

The online version contains supplementary material available at 10.1007/s10067-023-06760-z.

## Introduction

Continuous therapeutic treatment is important in the context of chronic autoimmune diseases. Treatment persistence, or the total time from initiation to discontinuation of therapy, depends upon drug efficacy, patient satisfaction, safety, and tolerability and is therefore regarded as a useful measure of overall drug effectiveness [1–4]. Moreover, suboptimal persistence tends to accompany greater patient morbidity and mortality [5], higher disease activity scores [6], and increased utilization of healthcare resources at greater cost [1, 7, 8]. Characterizing drug retention and discontinuation in different patient groups may provide further insight on persistence and, consequently, clinical outcomes [3].

Golimumab is a human monoclonal antibody directed against tumor necrosis factor–α (TNF-α), a potent pro-inflammatory cytokine whose signaling contributes to autoimmune diseases [9–11]. Randomized controlled clinical trials have established the efficacy and safety of golimumab treatment in patients with rheumatoid arthritis (RA), psoriatic arthritis (PsA), and ankylosing spondylitis (AS) [12–16]. Based on these results, golimumab has been approved in multiple countries and regions across the globe for the treatment of several rheumatic disorders. Long-term extensions of these trials reported retention rates of around 70% after five years of follow-up when golimumab was used as a 1^st^-line TNF-α inhibitor (TNFi) [17–19]. Long-term golimumab retention has also been shown to be favorable in several real-world cohorts, most notably in the BIOBADASER registry, which found a 38% retention at years 7 and 8 when golimumab was used as 1^st^-line therapy [20].

While golimumab retention was described previously in the pivotal Phase III clinical trials (RA: GO-BEFORE [12, 18], GO-AFTER [13, 21], GO-FORWARD [14, 19]; PsA: GO-REVEAL [15, 22]; AS: GO-RAISE [16, 17]), the results of these studies were not compared systematically by Kaplan–Meier analysis, an established standard for the interpretation of drug persistence data. This post-hoc analysis therefore aimed to evaluate golimumab treatment persistence for up to five years using pooled data from these pivotal Phase III studies.

## Participants and methods

### Study design and data source

Using data collected prospectively from five Phase III, randomized, placebo-controlled clinical trials, this post-hoc analysis evaluated drug retention of golimumab (either 50 mg or 100 mg administered subcutaneously every four weeks) for up to five years in participants with RA, PsA, and AS. Analyses of the five-year data from each of these individual studies have been reported previously [17–19, 21, 22].

Drug retention data from four of the five studies (GO-BEFORE, GO-FORWARD, GO-REVEAL, and GO-RAISE) were pooled for analysis of 1^st^-line golimumab therapy (i.e., treatment with golimumab in participants who had not previously been treated with a TNFi). Data from the remaining study (GO-AFTER) were used for analysis of 2^nd^- or further line (hereafter, 2^nd^-line) golimumab therapy (i.e., treatment with golimumab in participants who had previously received and discontinued at least one other TNFi [etanercept, adalimumab, or infliximab] for any reason).

Details of patient eligibility and study design were reported previously [12–19, 21, 22]. Key inclusion and exclusion criteria for each of the five studies are summarized in Supplemental Table 1. The similar eligibility criteria used for the four 1^st^-line golimumab therapy studies enabled pooled analysis in the present report. Briefly, these studies included adult patients who had an established diagnosis for the indication being studied without any other potentially confounding inflammatory diseases. In all studies except GO-AFTER, patients were naïve to TNFi therapy.GO-BEFORE was the only study to evaluate patients naïve to methotrexate (i.e., had not received more than 3 weekly doses of oral methotrexate for RA at any time).

### Statistical analysis

Baseline demographic and disease characteristics were described with summary descriptive statistics. Kaplan–Meier analyses were used to estimate the probability of golimumab persistence over time by study, indication, and line of therapy. Group differences in these analyses were evaluated by the log-rank test. Golimumab persistence rates were determined at baseline (Week 0), Year 1 (Week 52), Year 2 (Week 104), Year 3 (Week 156), Year 4 (Week 208), and Year 5 (Week 252) for all studies. A multivariate Cox regression model was used to analyze predictive factors related to overall golimumab persistence, which was built using the purposeful selection process and stepwise selection method. All analyses were performed using SAS version 9.4. No adjustments for multiplicity were made.

## Results

Participant demographic and baseline characteristics are summarized descriptively for the pooled 1^st^-line RA studies and by line of therapy (1^st^-line vs 2^nd^-line) (Table 1). Among the 2228 total participants enrolled in the five trials, 1797 participants received golimumab as 1^st^-line therapy (RA = 1050; PsA = 394; AS = 353) and 431 participants received golimumab as 2^nd^-line therapy (all with RA). Compared with the pooled 1^st^-line golimumab analysis cohort, participants that received 2^nd^-line golimumab had a longer disease duration (median of 9.2 years versus 3.7 years), were more likely to be female (78.7% versus 62.2%) and older than 50 years (61.5% versus 41.2%). In addition to having used and discontinued other TNFi therapies, more participants in the 2^nd^-line cohort had used prior corticosteroids (52.7% versus 41.3%) or methotrexate (66.1% versus 58.1%). The disproportionately higher proportion of RA participants in the 1^st^-line pooled studies (RA represents 58.4% of 1^st^-line studies) is likely to have influenced some of the observed baseline differences between the 1^st^- vs 2^nd^-line studies. Specifically, the higher proportion of females and prior medications (corticosteroids and methotrexate) seen in the pooled RA studies compared to the 1^st^-line pooled studies and 2^nd^-line RA study suggest that these observed differences in 2^nd^-line vs 1^st^-line therapy may be more indicative of indication as opposed to line of therapy. Supplemental Table 2, which further stratifies the participant baseline characteristics by study, shows the expected differences in gender and prior medications across the individual rheumatic diseases. In contrast, the notably shorter disease duration among participants in the pooled RA 1^st^-line studies (as well as the 1^st^-line compared to the 2^nd^-line RA study) suggest disease duration is more influenced by line of therapy than by indication. The same trend in disease duration was observed for each of the 1^st^-line rheumatic diseases compared to the 2^nd^-line RA study (Supplemental Table 2).

**Table 1:** Baseline demographic and disease characteristics for RA and 1^st^- versus 2^nd^-line therapy studies

Baseline Characteristics	1^st^-Line Pooled RA Studies *N* = 1050	1^st^-Line Pooled Studies *N* = 1797		2^nd^-Line^†^ RA Study *N* = 431	
Age — median (IQR), years	51.0 (42.0–57.0)	47.0	(38.0–56.0)	54.0	(46.0–63.0)
Age Group — *n* (%)					
≤ 50 years	524 (49.9)	1057	(58.8)	166	(38.5)
> 50 years	526 (50.1)	740	(41.2)	265	(61.5)
Gender — n (%)					
Female	859 (81.8)	1117	(62.2)	339	(78.7)
Male	191 (18.2)	680	(37.8)	92	(21.3)
Race — n (%)					
Caucasian	781 (74.4)	1422	(79.1)	376	(87.2)
Black	13 (1.2)	18	(1.0)	24	(5.6)
Asian	181 (17.2)	273	(15.2)	8	(1.9)
Other	75 (7.1)	84	(4.7)	23	(5.3)
Body Weight — median (IQR), kg	69.5 (59.5–81.4)	73.0	(62.0–87.0)	76.0	(65.0–90.3)
BMI — median (IQR), kg/m^2^	26.1 (23.0–29.9)	26.6	(23.3–30.5)	27.8	(24.1–32.6)
Smoking Status — n (%)					
Current Smoker	210 (20.0)	408	(22.7)	84	(19.5)
Prior Smoker	168 (16.0)	342	(19.0)	133	(30.9)
Non-smoker	672 (64.0)	1047	(58.3)	214	(49.7)
Disease Duration — median (IQR), years	2.7 (0.8–7.5)	3.7	(1.1–9.5)	9.2	(5.3–16.0)
Prior Medication — n (%)					
Corticosteroids	622 (59.2)	742	(41.3)	227	(52.7)
Methotrexate	780 (74.3)	1044	(58.1)	285	(66.1)
Patient VAS Pain Score — median (IQR)	6.4 (4.8–7.9)	6.6	(4.9–8.0)	6.9	(5.2–8.4)
Patient VAS GDA Score — median (IQR)	6.0 (4.4–7.8)	6.1	(4.6–7.8)	6.6	(5.0–8.2)

*RA*, rheumatoid arthritis; *IQR*, interquartile range; *BMI*, body mass index; *VAS*, visual analogue scale; *GDA*, global disease assessment

^†^2^nd^-line refers to any participant receiving golimumab after ≥ 1 line of another TNFi therapy. In the 2^nd^-line study (GO-AFTER), 115/461 (25%) participants received two and 43/461 (9%) received three TNFi therapies before enrollment. In the present analysis there were 431 participants who were treated with golimumab and had both a treatment-start date and a treatment-end date

Kaplan–Meier analysis was used to directly compare the modeled probability of golimumab treatment persistence over five years for the GO Phase III trials. Retention rates are presented by study, line of therapy, and indication in Table 2. In the pooled 1^st^-line therapy cohort (GO-BEFORE, GO-FORWARD, GO-REVEAL and GO-RAISE), golimumab retention was high across the five-year duration, with a probability of retention at Year 1 of 87.8% (95% CI: 86.2–89.2) and at Year 5 of 69.8% (95% CI: 67.6–71.9). Long-term treatment retention of 2^nd^-line golimumab (GO-AFTER) was lower than that of 1^st^-line golimumab, but remained favorable, with a probability of retention at Year 1 of 76.1% (95% CI: 71.8–79.9) and at Year 5 of 41.6% (95% CI: 36.8–46.3). Among the four individual 1^st^-line studies, rates were also similar, with retention at Year 1 ranging from 86.4% (GO-BEFORE) to 89.6% (GO-REVEAL) and from 67.0% (GO-BEFORE) to 71.8% (GO-FORWARD and GO-RAISE) at Year 5.

**Table 2:** Golimumab retention rates in 1^st^- and 2^nd^-line therapy studies

		Year 1	Year 2	Year 3	Year 4	Year 5	
Study and Indication	N	(Week 52)	(Week 104)	(Week 156)	(Week 208)	(Week 252)	*p*-value
1^st^-Line							
Pooled 1^st^-Line	1797	87.8 (86.2–89.2)	80.9 (79.0–82.6)	77.3 (75.3–79.2)	73.5 (71.4–75.5	69.8 (67.6–71.9)	0.2327^#^ 0.5106^†^
GO-BEFORE	616	86.4 (83.4–88.8)	78.4 (74.9–81.4)	74.8 (71.2–78.0)	70.8 (67.0–74.2)	67.0 (63.0–70.7)	-
GO-FORWARD	434	87.8 (84.3–90.5)	81.1 (77.1–84.5)	77.9 (73.7–81.5)	74.9 (70.5–78.7)	71.8 (67.2–75.8)	-
GO-REVEAL	394	89.6 (86.1–92.2)	84.3 (80.3–87.5)	79.4 (75.1–83.1)	74.9 (70.3–78.9)	70.1 (65.2–74.5)	-
GO-RAISE	353	88.4 (84.6–91.3)	81.3 (76.8–85.0)	78.7 (74.1–82.6)	75.0 (70.2–79.2)	71.8 (66.8–76.3)	-
Ankylosing Spondylitis	353	88.4 (84.6–91.3)	81.3 (76.8–85.0)	78.7 (74.1–82.6)	75.0 (70.2–79.2)	71.8 (66.8–76.3)	-
Psoriatic Arthritis	394	89.6 (86.1–92.2)	84.3 (80.3–87.5)	79.4 (75.1–83.1)	74.9 (70.3–78.9)	70.1 (65.2–74.5)	-
Rheumatoid Arthritis	1050	86.9 (84.8–88.8)	79.5 (76.9–81.8)	76.1 (73.4–78.5)	72.5 (69.7–75.1)	69.0 (66.1–71.8)	-
2^nd^-Line							
GO-AFTER	431	76.1 (71.8–79.9)	64.7 (60.0–69.0)	53.5 (48.7–58.1)	45.5 (40.7–50.1)	41.6 (36.8–46.3)	< 0.0001^‡^ < 0.0001^¶^

N denotes the number of participants at time zero (the time of first dose)

Year 1–5 data are presented as retention rate (%) with 95% confidence interval

*p*-values are based on the following log-rank tests:

^#^ retention rates across the four 1^st^-line studies

^†^ retention rates across the three indications studied in the 1^st^-line studies (RA, PsA, and AS)

^‡^ retention rates for pooled 1^st^-line studies vs 2^nd^-line RA study

^¶^ retention rates for pooled 1^st^-line RA studies vs 2^nd^-line RA study

**Fig. 1:**
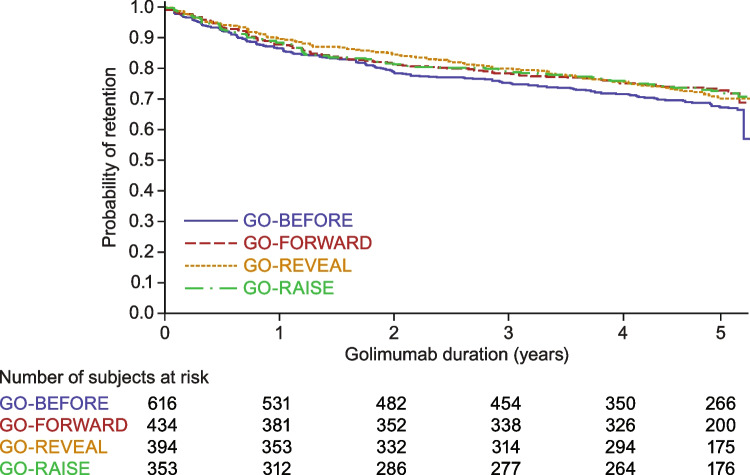
Probability of golimumab retention by study in 1^st^-line therapy

Results from the four 1^st^-line therapy trials were used to test whether conditions particular to these trials could contribute to differences in persistence at any time point (Fig. 1). Golimumab retention rates were consistent across each of the four 1^st^-line studies across their five-year durations (log-rank test: *p* = 0.2327).

**Fig. 2:**
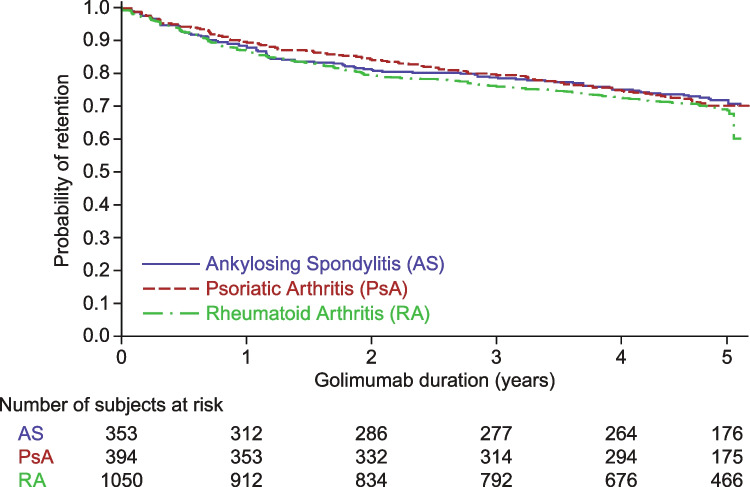
Probability of golimumab retention by indication in 1^st^-line therapy

Next, the modeled probability of golimumab treatment persistence was compared across the three rheumatic indications in the 1^st^-line therapy trials (Fig. 2), which also showed no differences in golimumab retention rates across these indications (log-rank test: *p* = 0.5106).

**Fig. 3:**
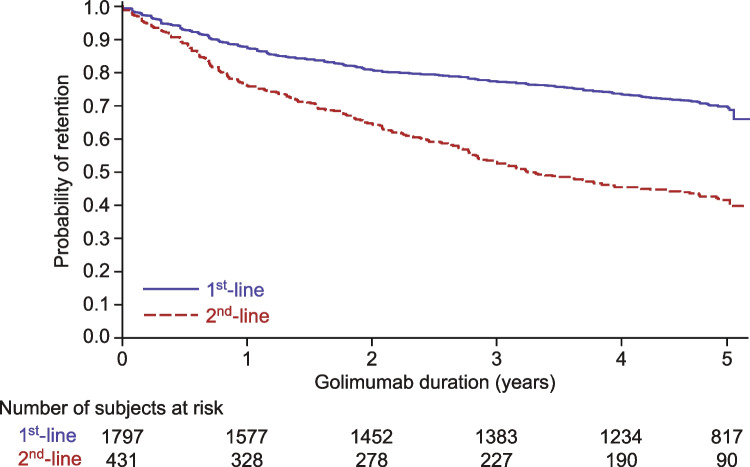
Probability of golimumab retention by line of therapy

In addition, the modeled probability of golimumab treatment persistence of the pooled cohort of participants that received golimumab as 1^st^-line therapy (GO-BEFORE, GO-FORWARD, GO-REVEAL, and GO-RAISE) was compared with those that received golimumab as 2^nd^-line therapy (GO-AFTER) (Fig. 3). Treatment retention was higher in participants using golimumab as a 1^st^-line therapy (pooled indications) than in those using it as 2^nd^-line therapy (log-rank test: *p* < 0.0001).

**Fig. 4:**
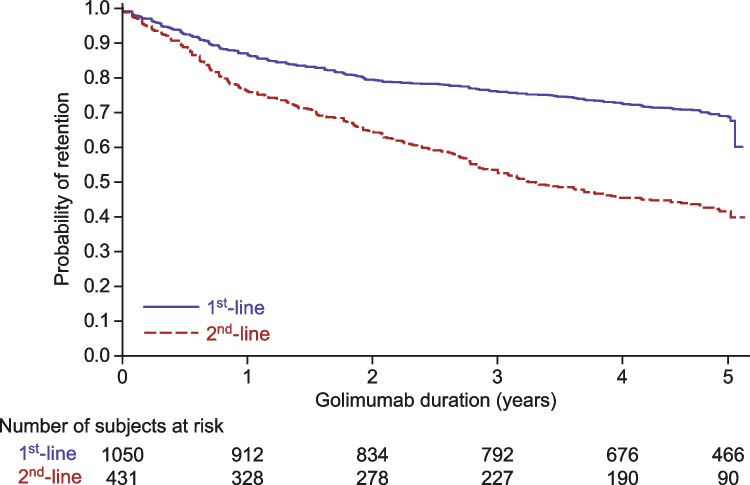
Probability of golimumab retention by line of therapy in participants with RA

Lastly, the modeled probability of golimumab treatment persistence of the pooled cohort of RA trial participants that received golimumab as 1^st^-line therapy was compared with that of the RA trial participants that received golimumab as 2^nd^-line therapy (Fig. 4). Treatment retention was higher in RA trial participants using golimumab as a 1^st^-line therapy (pooled RA trials) than in those using it as 2^nd^-line therapy (log-rank test: *p* < 0.0001).

The evaluation of predictive factors of golimumab treatment retention in the overall study population showed a significant association with race and concomitant methotrexate treatment in addition to indication and line of therapy (Supplemental Table 3). Of note, hazard ratios from the multivariate analysis suggest participants of Black race had a higher risk of not remaining on golimumab treatment compared to participants of Caucasian and Other races (Supplemental Table 4). In contrast, there was a higher likelihood of treatment persistence among participants with AS or PsA compared to RA as well as with AS compared to PsA. Participants receiving concomitant methotrexate also had a higher likelihood of treatment retention compared to those not receiving methotrexate.

## Discussion

Treatment persistence, or the total time from initiation to discontinuation of therapy, has been used as a surrogate measure of overall treatment success because it is influenced by drug efficacy, patient satisfaction, safety, and tolerability. The present study compared the treatment persistence of golimumab, a TNFi often taken for years by patients with chronic rheumatic disease, across multiple clinical trials in different rheumatic disease indications and lines of therapy. The results of this post-hoc analysis indicate highly favorable long-term retention of golimumab for trial participants with RA, PsA, or AS, typically around 70% at Year 5 when taken as a 1^st^-line biologic TNFi. No statistically significant differences in retention rates were observed across either 1^st^-line clinical trials (*p* = 0.2327) or disease indications (*p* = 0.5106). In comparison to the 1^st^-line trials, long-term golimumab retention was lower for participants with RA receiving 2^nd^-line therapy in the GO-AFTER trial (*p* < 0.0001). Similarly, among participants with RA, the golimumab retention rate was lower when used as 2^nd^-line therapy than when it was used as 1^st^-line therapy (*p* < 0.0001). Nonetheless it is noteworthy that over 40% of participants who already discontinued one or more TNFi therapies remained on golimumab at Year 5 in the GO-AFTER trial cohort.

The present study has a couple of important limitations. The disproportionately higher proportion of participants with RA compared to participants with PsA and AS in the 1^st^-line study cohort skew the overall baseline characteristic profile toward characteristics more typical of an RA patient population. Pooling the three RA 1^st^-line studies for comparison to the 2^nd^-line study (all RA) provides a clearer picture of the true differences between 1^st^- and 2^nd^-line therapy, highlighting longer disease duration with 2^nd^-line therapy. While the comparable probability of golimumab retention in each of the individual indications confirmed that it was appropriate to pool the 1^st^-line therapies for comparison to the 2^nd^-line therapies, inherent differences in disease characteristics and small subgroup sample sizes limited the evaluation of predictors of persistence to factors collected uniformly across all studies, including demographic and concomitant medications. Moreover, the lack of 2^nd^-line golimumab data from randomized controlled trials in PsA and AS limits generalizability beyond the RA patient populations.

Higher treatment persistence with 1^st^-line than with 2^nd^-line TNFi treatment is consistent with previous studies. Similar findings have been reported with 1^st^-line compared with 2^nd^-line golimumab treatment in patients in real-world clinical practice [20]. Lower retention rates are generally expected for patients or clinical trial participants who have already received and discontinued more than one drug in the same class because these individuals tend to have more established disease and are likely to be more difficult to treat [23–25]. The predictor analysis in the overall population of the current study showed a higher probability of treatment retention among participants with AS or PsA compared to RA as well as with AS compared to PsA. In addition to indication and line of therapy, the present study identified non-black race and concomitant medication with methotrexate as factors that promote treatment persistence. To improve treatment persistence rates for individuals with recalcitrant rheumatic disease, future studies comparing the specific reasons for discontinuation between 1^st^- and 2^nd^-line therapy groups, as well as factors associated with continued drug use particularly in 2^nd^-line patients, may be warranted. In addition, our data are limited to 2^nd^-line use of golimumab in participants with RA because this is the only population investigated for golimumab 2^nd^-line use in a randomized controlled trial. Whether golimumab 2^nd^-line retention rates differ across individuals with RA, PsA, or AS remains to be further explored, but real-world evidence suggests lower 2^nd^-line retention with RA [20]. Previous studies suggest that golimumab retention rates are at least as good as or better than those of other TNFi compounds in patients with rheumatic diseases [7, 20, 26–29].

A recent retrospective analysis of the BIOBADASER database reported rates of golimumab persistence lower than those found in our analysis of the GO Phase III trials [20]. The overall five-year retention rate in that study, including pooled data for approved indications (RA, PsA, and AS) and lines of therapy, was 43.9% (95% CI: 40.2–47.6), and approximately 56% for 1^st^-line therapy in particular [20]. The 3-to-5-year retention rates reported in other studies for golimumab as 1^st^ or greater line of therapy range from approximately 35% to 65% [29–35]. Patients in clinical practice can be more difficult to treat and may exhibit more disease comorbidities than the well-controlled clinical trial cohorts, possibly leading to lower retention. Rates observed in clinical trials are therefore not necessarily predictive of real-world drug persistence [3]. Nonetheless, the results from BIOBADASER remarkably showed that approximately 38% of the total adult rheumatology patient population remained on golimumab treatment for eight years after treatment initiation in a real-world setting [20].

Golimumab persistence reported in the literature is variable and is likely due to small sample sizes as well as differences in study designs and study populations (consisting of biologic naïve and biologic experienced, in addition to multiple lines of therapy), making inter study comparisons limited. For example, not all reports are consistent with the current finding that golimumab treatment persistence over 5 years does not differ between the approved rheumatic indications. In the BIOBADASER population, golimumab retention rates were shown to be higher among patients with AS or PsA than those with RA; however, this population consisted of a mixture of therapy lines that were roughly similar across the indications [20, 23]. Similarly, another study in a Spanish cohort reported that more patients with AS and PsA continued their first biologic drug than did patients with RA after a mean 3.8 years of follow-up [36]. A study of 328 individuals with RA, PsA, or AS at four centers in Greece reported overall high golimumab retention (62% with pooled lines of therapy) at three years, but rates were higher for patients with AS than for those with RA or PsA [35]. However, other shorter-term studies in Italy [37] and France [38] reported similar retention rates across indications after two years of golimumab treatment, particularly for 1^st^-line therapy. While some differences in real-world drug use could be attributed to differences in healthcare [39, 40], factors affecting retention rates in different indications are not yet fully understood and would be an important topic for future investigation, particularly in large cohorts with long follow-up duration.

## Conclusion

This post-hoc analysis of data collected prospectively from five Phase III clinical trials of golimumab in participants with rheumatic diseases presents, for the first time, comparative persistence results using Kaplan–Meier analysis. High treatment retention was seen across indications (RA, PsA, and AS), with at least two-thirds of patients estimated to remain on therapy five years after treatment initiation. Probability of long-term golimumab treatment retention with 2^nd^-line therapy, while lower than that of 1^st^-line therapy, also remained favorable with approximately 42% of participants estimated to remain on therapy at Year 5. Taken together, the present analysis supports the value of long-term golimumab use as 1^st^-line therapy in patients with rheumatic diseases (RA, PsA and AS) and as 2^nd^-line therapy in patients with RA.

### Supplementary Information

Below is the link to the electronic supplementary material.

Supplementary file1 (DOCX 52 KB)

## Data Availability

The data sharing policy, including restrictions, of Merck Sharp & Dohme LLC, a subsidiary of Merck & Co., Inc., Rahway, NJ, USA, is available at http://engagezone.msd.com/ds_documentation.php. Requests for access to the clinical study data can be submitted through the EngageZone site or via email to dataaccess@merck.com.
