# Supplementary material for: Long-term golimumab persistence: Five-year treatment retention data pooled from pivotal Phase III clinical trials in patients with rheumatoid arthritis, psoriatic arthritis, and ankylosing spondylitis
Source: Clin Rheumatol. 2023 Sep 26;42(12):3397–405. doi: 10.1007/s10067-023-06760-z (PMC10640568; doi:10.1007/s10067-023-06760-z)
Supplement: Supplementary file 1 — Supplementary file1 (DOCX 52 KB) [file 10067_2023_6760_MOESM1_ESM.docx]

**Supplemental Information for:**

**Long-term golimumab persistence: Five-year treatment retention data pooled from pivotal Phase III clinical trials in patients with rheumatoid arthritis, psoriatic arthritis, and ankylosing spondylitis**

Cindy L. J. Weinstein*^1^, Alan G. Meehan^1^, Jianxin Lin^1^, Steven D. Briscoe^1^, Marinella Govoni^2^

^1^Merck & Co., Inc., Rahway, NJ, USA

^2^MSD Italy, Rome, Italy

Corresponding author:

Dr. Cindy L. J. Weinstein

126 East Lincoln Avenue

Rahway, NJ USA 07065-0900

[cindy.l.weinstein@merck.com](mailto:cindy.l.weinstein@merck.com)

ORCID iD: 0000-0003-4229-4644

**Supplemental Table 1.** Inclusion and exclusion criteria for 1^st^- and 2^nd^-line therapy studies

| **Study** | **Population** | **Key Inclusion Criteria** | **Key Exclusion Criteria** |
| --- | --- | --- | --- |
| GO-BEFORE^12^ | RA  MTX-naïve | - Adults ≥ 18 years. - Diagnosis of RA for ≥ 3 months. - Active RA (defined as persistent disease activity with ≥ 4 swollen and ≥ 4 tender joints at screening and baseline). - MTX-naïve (defined as ≤ 3 weekly doses of MTX for RA at any time). - Must have met ≥ 2 of the following criteria:   - CRP ≥ 1.5 mg/dL at screening or ESR ≥ 28 mm at either screening or within the first hour baseline   - Morning stiffness of ≥ 30 minutes at screening and baseline.   - Bone erosion by x-ray and/or MRI.   - Anti-CCP antibody-positive or RF-positive at screening. | - Inflammatory diseases other than RA that might have confounded the evaluation of benefit from golimumab therapy. - Previous treatment with TNFi therapy at any time; DMARDs/systemic immunosuppressives; intra-articular, intramuscular, or intravenous corticosteroids; or anakinra within 4 weeks prior to the first study dose. |
| GO-FORWARD^14^ | RA  Inadequate response to MTX | - Adults ≥ 18 years. - Diagnosis of RA for ≥ 3 months. - Active RA (defined as persistent disease activity with ≥ 4 swollen and ≥ 4 tender joints at screening and baseline). - Must have met ≥ 2 of the following criteria:   - CRP ≥ 1.5 mg/dL at screening or ESR ≥ 28 mm at either screening or within the first hour baseline   - Morning stiffness of ≥ 30 minutes at screening and baseline.   - Bone erosion by x-ray and/or MRI.   - Anti-CCP antibody-positive or RF-positive at screening. - Must have tolerated MTX at a dose of ≥ 15 mg/week for ≥ 3 months prior to screening and have received MTX at a dose of ≥ 15 mg/week and ≤ 25 mg/week and stable for ≥ 4 weeks prior to screening. - Subjects who were receiving stable low doses of oral corticosteroids for ≥ 2 weeks prior to the first administration of study agent and/or stable doses of NSAIDs for ≥ 2 weeks prior to the first administration of study agent. | - Inflammatory diseases other than RA that might have confounded the evaluation of benefit from golimumab therapy. - Previous treatment with TNFi therapy at any time; DMARDs/systemic immunosuppressives; intra-articular, intramuscular, or intravenous corticosteroids; or anakinra within 4 weeks prior to the first study dose. |
| GO-REVEAL^15^ | PsA  Inadequate response to DMARD/NSAID | - Adults ≥ 18 years. - Diagnosis of PsA. - Active psoriasis defined by ≥ 3 swollen and ≥ 3 tender joints, negativity for RF, and the presence of plaque psoriasis. - Active psoriasis despite therapy with DMARDs or NSAIDs. | - Inflammatory diseases other than PsA that might have confounded the evaluation of benefit from golimumab therapy. - Previous treatment with TNFi therapy at any time, rituximab, natalizumab, or cytotoxic agents. |
| GO-RAISE^16^ | AS  Inadequate response to DMARD/NSAID | - Adults ≥ 18 years. - Diagnosis of AS for ≥ 3 months. - BASDAI score ≥ 4 and spinal pain assessment score ≥ 4 on a visual analog scale. - Inadequate response to current or previous NSAIDs or DMARDs. | - Inflammatory diseases other than AS that might have confounded the evaluation of benefit from golimumab therapy. - Complete ankylosis of the spine. - Previous treatment with TNFi therapy at any time, rituximab, natalizumab, cytotoxic agents, systemic immunosuppressives, or alefacept or efalizumab within 3 months before first administration of study drug. |
| GO-AFTER^13^ | RA  Previous use of ≥ 1 other TNFi therapy | - Adults ≥ 18 years. - Diagnosis of RA for ≥ 3 months. - Active RA (defined as persistent disease activity with ≥ 4 swollen and ≥ 4 tender joints). - Documentation of previous treatment with at least one dose of TNFi agent (etanercept, adalimumab, or infliximab), the last dose of which must have been given ≥ 12 weeks (infliximab) or ≥ 8 weeks (adalimumab or etanercept) prior to first administration of study agent. Participants could have discontinued these agents for any reason. - The only DMARDs permitted during the study were MTX, sulfasalazine, and hydroxychloroquine (alone or in combination). - Low doses of oral corticosteroids for ≥ 2 weeks prior to first administration of the study agent and/or stable doses of NSAIDs for ≥ 2 weeks prior to first administration of study agent were also permitted. | - Inflammatory diseases other than RA that might have confounded the evaluation of benefit from golimumab therapy. - Serious adverse reaction to a previous TNFi (judged by the investigator) - Previous treatment with rituximab, natalizumab, cytotoxic agents, anakinra within 4 weeks prior to the first study dose, alefacept or efalizumab within 3 months prior to the first study dose |

Abbreviations: AS, ankylosing spondylitis; BASDAI, Bath Ankylosing Spondylitis Disease Activity Index; CCP, cyclic citrullinated peptide; CRP, c-reactive protein; DMARD, disease-modifying antirheumatic drug; ESR, erythrocyte sedimentation rate; MTX, methotrexate; NSAID, nonsteroidal anti-inflammatory drug; PsA, psoriatic arthritis; RA, rheumatoid arthritis; RF, rheumatoid factor.

**Supplemental Table 2.** Baseline demographic and disease characteristics for 1^st^-line RA, AS and PsA versus 2^nd^-line RA studies

| **Baseline Characteristics** | **RA 1^st^-Line**  **N=1050** | **AS 1^st^-Line**  **N=353** | **PsA 1^st^-Line**  **N=394** | **RA 2^nd^-Line**  **N = 431** |
| --- | --- | --- | --- | --- |
| Age — median (IQR), years | 51.0 (42.0-57.0) | 38.0 (29.0-47.0) | 47 (39.0-54.0) | 54.0 (46.0–63.0) |
| Age Group — n (%) |  |  |  |  |
| ≤ 50 years | 524 (49.9) | 292 (82.7) | 241 (61.2) | 166 (38.5) |
| > 50 years | 526 (50.1) | 61 (17.3) | 153 (38.8) | 265 (61.5) |
| Gender — n (%) |  |  |  |  |
| Female | 859 (81.8) | 100 (28.3) | 158 (40.1) | 339 (78.7) |
| Male | 191 (18.2) | 253 (71.7) | 236 (59.9) | 92 (21.3) |
| Race — n (%) |  |  |  |  |
| Caucasian | 781 (74.4) | 259 (73.4) | 382 (97.0) | 376 (87.2) |
| Black | 13 (1.2) | 3 (0.8) | 2 (0.5) | 24 (5.6) |
| Asian | 181 (17.2) | 85 (24.1) | 7 (1.8) | 8 (1.9) |
| Other | 75 (7.1) | 6 (1.7) | 3 (0.8) | 23 (5.3) |
| Body Weight — median (IQR), kg | 69.5 (59.5-81.4) | 75.3 (64.0-87.0) | 84.0 (70.0-96.8) | 76.0 (65.0–90.3) |
| BMI — median (IQR), kg/m^2^ | 26.1 (23.0-29.9) | 25.7 (22.5-29.1) | 28.6 (25.1-33.0) | 27.8 (24.1–32.6) |
| Smoking Status — n (%) |  |  |  |  |
| Current Smoker | 210 (20.0) | 127 (36.0) | 71 (18.0) | 84 (19.5) |
| Prior Smoker | 168 (16.0) | 72 (20.4) | 102 (25.9) | 133 (30.9) |
| Non-smoker | 672 (64.0) | 154 (43.6) | 221 (56.1) | 214 (49.7) |
| Disease Duration — median (IQR), years | 2.7 (0.8-7.5) | 5.6 (1.7-13.1) | 5.1 (1.8-10.3) | 9.2 (5.3–16.0) |
| Prior Medication — n (%) |  |  |  |  |
| Corticosteroids | 622 (59.2) | 57 (16.1) | 63 (16.0) | 227 (52.7) |
| Methotrexate | 780 (74.3) | 72 (20.4) | 192 (48.7) | 285 (66.1) |
| Patient VAS Pain Score — median (IQR) | 6.4 (4.8-7.9) | 7.6 (6.4-8.6) | 5.7 (4.0-7.5) | 6.9 (5.2–8.4) |
| Patient VAS GDA Score — median (IQR) | 6.0 (4.4-7.8) | 7.1 (6.0-8.3) | 5.2 (3.9-7.0) | 6.6 (5.0–8.2) |

Abbreviations: RA, rheumatoid arthritis; AS, ankylosing spondylitis; PsA, psoriatic arthritis; IQR, interquartile range; BMI, body mass index; VAS, visual analogue scale; GDA, global disease assessment.

 2^nd^-line refers to any participant receiving golimumab after ≥ 1 line of another TNFi therapy. In the 2^nd^-line study (GO-AFTER), 115/461 (25%) participants received two and 43/461 (9%) received three TNFi therapies before enrollment. In the present analysis there were 431 participants who were treated with golimumab and had both a treatment-start date and a treatment-end date.

**Supplemental Table 3.** Multivariate analysis of predictive factors for golimumab retention*

| **Effect** | **DF** | **P-value** |
| --- | --- | --- |
| Race Group | 3 | 0.0005 |
| Concomitant Medication - Methotrexate | 1 | <.0001 |
| Indication | 2 | <.0001 |
| First/Second Line Therapy | 1 | <.0001 |

Abbreviations: DF; degrees of freedom.

*Purposeful selection method used to build a multivariable Cox model began with a careful univariable analysis of each independent variable. Using these univariable analyses candidates for the initial multivariable model were identified as any variable whose univariable test had a p-value < 0.15. The stepwise selection method was used to select the variables in the final model.

**Supplemental Table 4.** Multivariate analysis (hazard ratio) of predictive factors for loss of golimumab retention

| **Factor** | **Comparison** | **Hazard Ratio** | **95% CI** |
| --- | --- | --- | --- |
| Race Group | Asian vs Black | 0.45 | (0.29, 0.69) |
| Race Group | Asian vs Caucasian | 0.94 | (0.73, 1.20) |
| Race Group | Asian vs Other | 0.73 | (0.50, 1.07) |
| Race Group | Black vs Caucasian | 2.09 | (1.44, 3.04) |
| Race Group | Black vs Other | 1.64 | (1.02, 2.62) |
| Race Group | Caucasian vs Other | 0.78 | (0.57, 1.07) |
| Concomitant Medication - Methotrexate | Yes vs No | 0.42 | (0.36, 0.49) |
| Indication | AS vs PsA | 0.77 | (0.59, 1.02) |
| Indication | AS vs RA | 0.54 | (0.42, 0.69) |
| Indication | PsA vs RA | 0.70 | (0.56, 0.87) |
| First/Second Line Therapy | First line vs Second line | 0.54 | (0.46, 0.65) |

Abbreviations: CI, confidence interval.

Multivariate Cox regression analysis was used to evaluate the effect of several factors (covariates) on golimumab retention. In this context, the hazard ratio (HR) for each covariate indicates the probability of loss of golimumab retention in the first subgroup vs the second subgroup (i.e., a HR > 1 indicates the first subgroup of covariate has a higher probability of loss of golimumab retention).
